# New Communication Technology and the Elderly: A Study on the Continuous Use of the Extreme Edition APP for Middle-Aged and Senior Citizens

**DOI:** 10.3390/bs14121126

**Published:** 2024-11-24

**Authors:** Zeheng Liang, Yixin Xie, Ran Xu, Peng Gu

**Affiliations:** 1 School of Communication, Soochow University, Suzhou 215031, China; liangzeheng1316@163.com (Z.L.); x1xin818@163.com (Y.X.); 2 Domestic Cooperation and Development Office, Soochow University, Suzhou 215031, China; xrdxx@suda.edu.cn

**Keywords:** cognitive–emotional–behavioral, middle-aged and older adults, extreme edition app, addiction model

## Abstract

Rapidly changing digital technologies are reconfiguring the way human society lives, indicating that more and more middle-aged and older adults will lead a digital life in the future. Whether digital technology for today can effectively improve the quality of digital life of this cohort is the focus of this study. This study proposed a “cognitive–emotional–behavioral” model and situated the use of the Extreme Edition App as a cross-sectional research object. The study also explored the relationship between middle-aged and older adults’ perceptions of the benefits of cash subsidies, the pleasure and worry generated by the use of the app, and their continued use of the app. It has become a fact that human beings are walking side by side with digital technology; digital technology still moves forward and upward. Thus, it is forward-looking to pay attention to the digital life adaptation of the current middle-aged and older groups. A total of 1200 valid questionnaires were obtained, and regression analysis showed that (1) the more comprehensive and in-depth the cohort’s knowledge of the benefits of cash subsidies is, and the more sustainable their continuous use of the Extreme Edition App is, the more pleasure they experience, and the less worry they feel during its use. (2) The more pleasure middle-aged and older adults feel while using the Extreme Edition App, the more likely they are to continue using it. Conversely, the more worry they feel, the less likely they are to maintain its use. (3) Emotions generated during the use of the Extreme Edition App mediate the relationship between this cohort’s perceptions of cash subsidy benefits and their continued-use behavior.

## 1. Introduction

In the current era of globalization, population aging has become a global phenomenon. In 2023, the 78th session of the United Nations reported that “Europe, the United States, North America, Australia, New Zealand, and most of East and Southeast Asia currently have the highest levels of population aging”. [1] Data from China’s National Bureau of Statistics show that in 2021, China’s population aged 60 and above reached 267 million, accounting for 18.9% of the total population, of which 200 million were aged 65 and above and accounting for 14.2% of the total population [2]. Meanwhile, the 53rd Statistical Report on Internet Development in China pointed out that as of December 2023, the proportion of internet user groups aged 50 and above increased from 30.8% in December 2022 to 32.5%, implying that the internet has penetrated further into the middle-aged and older adult cohort [3].

We must understand that “aging” is an inevitable process for all human beings, and that the understanding of “old age” is not only related to older adults but also to not-so-old people (middle-aged people, young people, and even children and adolescents). Therefore, in the current digital era, this study intends to pay close attention to the process of digital media mastery and enhancement of older adults’ abilities and to continue to use digital technology to construct a digital-friendly social atmosphere efficiently for older adults so that every one of them can connect smoothly with the developments of the times and live with dignity, hope, and quality.

QuestMobile released the characteristics of apps on the TOP10 list of app usage preferences of “silver-haired people” (over 50 years old) and found that nine apps were characterized by “cash incentives.” [4] On the surface, the Extreme Edition App attracts middle-aged and older adults with its money-making features. However, in reality, it contains the hidden worry that their data might be “harvested” by traffic.

Based on these findings, this study first uses a faster, smaller, simpler, and cash-motivated app as the research sample to apply to a cross-sectional study of the digital living environment of Chinese middle-aged and older adults. Second, this study selects China’s 50-plus age group as the target group, which includes older adults over 65 (the international threshold of older age) and the middle-aged over 50 (China’s minimum retirement age) to explore the social sustainability of improving and optimizing the digital literacy of this cohort in China. Finally, this study takes “cognition–emotion–behavior” as the research logic, mainly revealing the relationship between middle-aged and older adults’ understanding of the benefits of the cash subsidy of the Extreme Edition App and their continuous use of it, and at the same time, introducing two emotions, pleasure and worry, as mediating variables to examine the role of emotion in the cognitive–behavioral relationship.

## 2. Literature Review

### 2.1. Cognitive–Emotional–Behavioral Model

The cognitive–emotional–behavioral model is derived from the attitude–behavior logic of the theory of planned behavior (TPB) [5] and the cognitive–emotional–intentional model in cognitive psychology. First, as a stable and enduring psychological concept [6] that can influence and predict human behavior effectively, the concept of attitude is often applied in projects that study human beings making decisions or engaging in particular behaviors. In addition, in the study of behavioral motivation, attitudes and their components can be used to explain the relationships between various human behaviors and, as a result, predict future behaviors [7,8]. Second, in consumer behavior, Wilkie stated that human behavior is composed of three dimensions: mental, emotional, and physical. Among them, the psychological and emotional dimensions were further subdivided into cognition, emotion, and intention in Rosenberg and Hovland’s study. Based on the former study, Kim (2013) argued that the concept of attitude has been widely described or analyzed as cognitive, affective, and intentional attitudes [9], which corresponds to the three dimensions of the “cognitive–affective–intentional” model for predicting individual behavior.

To sum up, this study integrates the three dimensions of attitude (cognitive, affective, and intentional) into the logic of attitude–behavior to arrive at the cognitive–emotional–intentional–behavioral model and simplifies the stage of intentionality to arrive at the cognitive–emotional–behavioral model. The simplification of the intention stage is mainly due to the following two reasons: on the one hand, intention is a behavioral tendency based on cognition and emotion, and in the TPB, intention can predict the occurrence of behaviors directly, which means that intention can be reflected directly in actual behaviors. On the other hand, some scholars have pointed out that “all observable behaviors are influenced by a combination of emotion and cognition” [10]. Hence, to examine the continuous use behaviors of middle-aged and older adults more directly and make the whole study more realistic and scientific, the intention stage was omitted. In the cognitive–emotional–behavioral model, cognition is a thinking process based on knowledge and experience or rational calculation. Emotion is the response to a specific thing formed based on cognition, in which emotion can be divided into positive and negative emotions. Behavior represents the actions that individuals habitually perform based on the first two.

This study examines the continuous use of the Extreme Edition App by middle-aged and older adults based on the cognitive–emotional–behavioral model, in which the cognitive stage refers to middle-aged and older adults’ perceptions of the benefits of the special feature of the “cash subsidy” during their continuous use of the app. The four phases of the addiction model (trigger, action, reward, and additional input) are used to form the four dimensions of the “cash subsidy” variable. The affective stage refers to the positive emotions–pleasure and negative emotions–worry that middle-aged and older adults experience when using the Extreme Edition App. The behavioral stage refers to the actual behavior of the users when using the app.

### 2.2. Middle-Aged and Older Adults’ Continuous Internet Use

Social media platforms are becoming popular among middle-aged and older adults owing to the advantages of continuously lowering the technological threshold, incorporating easy-to-use functions, and offering an abundance of information. Some researchers have pointed out that the main activities that older adults engage in on social networking sites include presenting themselves, viewing information about others, and keeping in touch with friends and family [11,12,13]. In addition, previous studies have suggested that older people’s use of social networks reduces social isolation and loneliness [14] while leading to greater life satisfaction and quality of life [15,16].

However, some studies have suggested that older adults are more likely to become addicted to social networking sites than younger adults [17], leading to more severe depression and anxiety. Leist’s study (2013) found that older adults’ use of social networking sites is influenced by factors such as social connectedness, social support, and recreation [18]. Chew et al. (2023) summarized the factors that influence older adults’ use of social networking sites, including the perceived usefulness of the technological dimension, trust in the individual dimension, and social influence of the social dimension [19].

Reuben Ng and Nicole Indran (2023) explored how older TikTok users express self-perceptions of aging in their videos. Their study found that older TikTok users tend to portray themselves positively and favorably in a variety of areas, and that the topics of their videos correlate significantly with physical functioning and social interactions. They also found that TikTok can be a platform for improving the perceptions of older adults [20].

With the advance of the internet era, the digital life pattern has become more and more obvious, and the middle-aged and older adult groups are both passively involved in and actively integrated into digital torrents, and digital technology products and services tailored to middle-aged and older adults have gradually emerged, such as mobile intelligent communication devices and related apps specially designed for this group, and related research has gradually followed up. Marcel Heerink et al. (2008) found that the acceptance of robotics by older adults is influenced by various factors. Among them, perceived enjoyment is an important factor that significantly influences older people’s intention to use robotic systems [21]. The 2012 IEEE Research Symposium on Humanities and Engineering found that “perceived value” was significant for older people’s intention to use smartphones, followed by facilitating conditions and effort expectations [22].

Qi Ma et al. (2016), through a questionnaire survey and interviews with 120 Chinese older adults aged over 55 years, found that age and education level were the most important personal factors influencing smartphone acceptance among older Chinese adults, while intrinsic motivation also played an important role [23]. In addition, access to external help plays a crucial role in the acceptance of smartphones by older adults [24]. Rakibul Hoque and Golam Sorwar’s (2017) extended model based on the UTAUT model influences the key factors of the adoption of mobile health (mHealth) services among older adults. The study found that factors such as performance expectations, effort expectations, social influences, technology anxiety, and resistance to change largely determine older adults’ willingness to use mHealth services [25]. Golam Sorwar and Junghee Cho (2023) explored the changes in older adults’ receptivity toward digital technology during the 2019 coronavirus pandemic and the factors influencing them. It was found that more than one-third of 1171 65-year-olds in South Korea reported positive changes in their perceptions of digital technology, with self-efficacy, digital networks, accessibility of digital devices, and perceived health considerably influencing older adults’ perceptions of digital technology [26].

### 2.3. Extreme Edition App and “Cash Subsidy” Benefit Perception

In China’s app market, 2019 was the first year of the surge of the Extreme Edition App. According to an insight report about the Extreme App released by QuestModel, the Extreme Version is designed to adapt to the lower-tier users who have low configuration of mobile phone. It is aimed at very low-age and older users, is easier to operate, and costs less in terms of user education [27]. Compared with the ordinary version of the app, the Extreme Edition App offers the following service characteristics: speed, small size, simple pages, and cash incentives. Therefore, this study defines the app as an application with the main feature of “users complete the corresponding tasks on the platform to win ‘gold coins’ and realize withdrawing them into CNY”.

According to the professional app ranking list released by Cicada Master, the daily downloads of the Extreme Edition App for Jingdong, Baidu, and Kwai are in the range of 30,000–40,000, and such high-frequency and large-scale downloads show the commercial potential of the Extreme Edition App. Therefore, many internet head enterprises have drawn a commercial map for Extreme Edition. The “Gold Coin Activity Rules” of Today’s Headlines Extreme Edition state that, to cultivate good reading and information-acquisition habits among users and the general public, a certain number of gold coins or points will be awarded to users who have completed specific tasks (according to the names of different operating systems, hereinafter collectively referred to as “gold coins”) and exchanged for a variable amount of cash. Such tasks include, but are not limited to, reading articles, viewing videos, walking, sleeping, eating, answering questions, planting trees, and growing vegetables.

The altruistic marketing of the Extreme Edition App, which has simple tasks and helps users earn money easily, has successfully attracted middle-aged and older people. First, middle-aged and older-aged groups have certain difficulties with digital technology and tend to dismiss it, so they pursue simpler and better-operated digital products. However, the interface of the Extreme Edition App is simple, the functions are streamlined, and the operation is extremely convenient. Second, under the banner of “making money by watching videos”, the app further satisfies the personal needs of middle-aged and older people for sustainable economic value creation. Finally, the personalized algorithm of the app recommends content, which allows middle-aged and older people to obtain spiritual comfort during the process of acquiring knowledge and information.

Although middle-aged and older people use the Extreme Edition App due to their unfamiliarity with digital products and digital technology, obtaining money via the app involves behaviors such as bank card binding, which may stimulate their risk-defense mechanism. Overall, the use of the app can bring pleasure to middle-aged and older people. However, it also generates a certain sense of worry.

To measure the effects of the “cash subsidy” function of the Extreme Edition App on middle-aged and older people’s continuous use of the app, this study introduces the four phases of the addiction model: triggering use, taking action, variable reward, and additional investment to construct the four dimensions of “cash subsidy”. The addiction model mainly helps enterprises cultivate users’ habit of using a product, and once users can use the product habitually without relying on external motivation, they will not easily change the product [28]. Based on this, this study proposes the following hypotheses:

**H1a:**  *Middle-aged and older people’s perceptions of the benefits of cash subsidies play a significant positive role in influencing their feelings of pleasure when using the Extreme Edition App*.

**H1b:**  *Middle-aged and older people’s perceptions of the benefits of cash subsidies have a significant positive influence on their feelings of worry during the use of the Extreme Edition App*.

#### 2.3.1. Trigger Use

Trigger use includes external and internal triggers. External triggering refers to guiding users to take the next step by introducing information in all aspects of their lives. The Extreme Edition App reminds middle-aged and older people of the dynamics, benefits, and value of this app through advertising posters, advertising videos, and other content. Internal triggering is to remind users to take the next step toward the product through various associations with the product, such as thoughts or emotions in the user’s memory [28]. Most members of this cohort are at the stage of retirement or close to retirement, and the insecurity caused by the sudden decrease in income is one type of internal trigger. The cash subsidy function in the app constantly stimulates middle-aged and older people’s interest in increasing their incomes. Based on this, we propose the following hypothesis:

**H2a:**  *Willingness to use triggered by cash subsidies plays a significant positive role in influencing middle-aged and older people’s continued use of the Extreme Edition App*.

#### 2.3.2. Action Taken

According to the behavioral model established by Fogg, people cannot act without sufficient motivation, the ability to accomplish a behavior, and a trigger that motivates them to take action [29]. In terms of motivation, Fogg believed that three core sets of motivations drive people to take action: the pursuit of pleasure and the avoidance of pain, the pursuit of hope and the avoidance of fear, and the pursuit of recognition and the avoidance of rejection. The middle-aged and older-aged groups have relatively more free time after retirement and are perhaps easily trapped in a mood of emptiness, loneliness, or even frustration. Growing age and deteriorating physical condition are more likely to produce the shadow of anxiety and fear, and retirement means separation from mainstream society, which creates social and personal values and is more likely to induce negative emotions of bitterness and loss. Therefore, middle-aged and older people must discover new ways of social participation and self-identity.

With the help of the Extreme Edition App, middle-aged and older people cannot only understand their health status through the content of the medical tips but can also be encouraged to record their lives, publish their video works, and gain quantifiable and visible recognition such as likes and comments, which makes them feel happy and brings fulfillment to their lives. Moreover, they can also earn real money in the form of “cash subsidies”, a new way of social participation that effectively relieves the uneasiness of leaving the workplace.

In terms of mobility, middle-aged and older people have enough time to use the app. Based on the convenient operation of mobile terminal equipment, the use of the app requires no physical strength. There is a low requirement to earn cash rewards by completing app tasks, such as browsing short videos, checking in, drinking water, and sleeping. The digital technology threshold of this app is decreasing, and the operation is becoming increasingly convenient. Finally, friends and relatives around middle-aged and older people are also using this app, and the herd psychology effect of its use is obvious. Overall, the app itself distinguishes itself from the original version of the app in that it simplifies many functions, configurations, and memory, helping to reduce the friction of the use process and further promoting this cohort to take action. Based on this, the following hypotheses are proposed in this study:

**H2b:**  *The motivation and ability to take action to obtain cash subsidies play a significant positive role in influencing the continued use of the Extreme Edition App by middle-aged and older people*.

#### 2.3.3. Variable Rewards

The main reason people use products is to satisfy their personal needs. Reward is one of the best stimuli to motivate people to use a certain product more, and it includes three main aspects: social, prey, and self [28]. Social rewards refer to interpersonal rewards that people obtain from interacting with others through a product. For example, middle-aged and older people obtain interpersonal and cash rewards by uploading video content and obtaining likes and comments via the Extreme Edition App. Prey rewards refer to specific resource information people obtain from a product. In the app, users receive different shares of cash subsidies by continuously completing different tasks. Meanwhile, regarding the algorithm’s personalized recommendation, content that meets user preferences gradually captures user attention. Thus, users are willing to use the app and complete tasks even if they do not have cash subsidies. Self-reward refers to the sense of achievement and control that a product offers. Middle-aged and older people experience a double sense of achievement in the process of using the app, in which they complete daily tasks one by one and earn more cash subsidies. In addition, a circular progress bar will appear on the pages of watching videos, reading articles, browsing advertisements, etc., in the app, which reminds users whether the cash subsidy rewards of each segment are acquired or not, and urges the users to complete the tasks in the circular progress bar to obtain the maximum amount of cash subsidies. The endless forms of tasks and uncertain cash subsidy rewards associated with each use of the app constantly maintain the long-term interest of users. Based on this, this study proposes the following hypotheses:

**H2c:**  *The sense of achievement gained from variable cash subsidies plays a significant positive role in influencing middle-aged and older people’s continued use of the Extreme Edition App*.

#### 2.3.4. Additional Input

The sense of gain and achievement formed in the three stages of triggering use, taking action, and implementing variable rewards makes users expect more cash subsidies and engage in additional actions [28]. This extra input action is manifested in two aspects. One of these approaches is loading different trigger paths because each trigger path has its own cash subsidy. In the other aspect, the app indicates the “input” window. For example, the taskbar requires users to browse live content, and users can shop directly during live broadcasts. Middle-aged and older people can also obtain cash subsidies by completing browsing tasks and shopping behaviors during the live broadcast of the app. Based on this, this study proposes the following hypothesis:

**H2d:**  *The additional input triggered by cash subsidies has a significant positive effect on the continuous use of the Extreme Edition App by middle-aged and older people*.

### 2.4. Pleasure and Worry

Emotions are ubiquitous and unique and occur in the daily lives of different individuals. They influence their beliefs and attitudes and guide their thinking, decision-making, and behavior [30,31].

Among positive emotions, pleasure is the most common and important. Pleasure refers to the sense of pleasure and interest that users obtain when using a particular technology or information system. According to the consumer behavior theory, it is human nature to seek pleasurable experiences [32]. In 1992, Davis introduced entertainment in the TAM, which is considered the entertainment and pleasure that users can feel during the mere use of technology without considering the actual performance brought by the use of the technology [33]. Moon and Kim (2001), in a study explaining user behavior when using the World Wide Web, introduced entertainment and pointed out that it plays a positive role in users’ continued use of the technology [34]. Kim et al. (2007) pointed out that users use smartphones based on the motivational effects provided by the state of satisfying both instrumental and hedonic needs [35], and it can be found that in the discussion of continuous use behavior, research has emphasized the benefits of hedonicity and its usefulness to users.

From the existing research literature, when studying users’ behavior when using information systems, scholars usually introduce variables about users’ motivation, which is divided into external and internal motivation. External motivation emphasizes the actual results or rewards obtained by the user, while internal motivation explores the user’s need for self-efficacy and emotional needs such as pleasure and interest. Lin (2005) found that entertainment plays an important role in positively influencing users to keep visiting a website, and it is a strong internal motivation [36]. Pleasure is one kind of internal motivation, and emotion is one of the internal motives that triggers the cycle of the addiction model. Therefore, to enhance the explanatory power of the research model, the variable of pleasure is introduced and defined as the pleasure that middle-aged and older people feel during the process of using the app.

The opposite of positive emotions is negative emotions, which are generally reflected in the feelings of worry, fear, and anxiety that individuals display when they encounter risks [37]. As a typical negative emotion, worry is generally triggered when there is a significant or personally relevant threat, while the sense of worry is a key driver in awakening an individual’s motivation to protect [38]. Beaudry and Pinsonneault (2010) introduced emotions to explore their impact on first-time IT use, where negative emotions were categorized as loss and deterrence emotions [39]. Deterrent emotions, including worry and anxiety, were found to be negatively related to IT use. As “slow walkers” in the digital age, middle-aged, and older people have a limited understanding of digital products and are, therefore, more likely to worry about unknown risks, especially when using the Extreme Edition App, which involves monetary transactions. Therefore, middle-aged and older people will choose to reduce or stop using the app to ensure their safety in the event of potential threats such as privacy and economic risks. In summary, this study proposes the following hypotheses regarding the association between the emotions generated during the use of the app and its continuous use by middle-aged and older people:

**H3a:**  *The pleasure generated during the use of the Extreme Edition App by middle-aged and older people positively influences their continued use*.

**H3b:**  *The feeling of worry generated during the use of the Extreme Edition App by middle-aged and older people has a negative influence on their continued use*.

In addition, Wu et al. (2020) showed that pleasure plays an intermediary role in the relationship between the two types of online store features (informative and entertainment) and consumer proximity behavior [40]. Oztekin (2024) pointed out that depression, anxiety, and worry play a mediating role between internet addiction and behavioral engagement [41]. Based on this, the following hypotheses are proposed to address the question of whether there is a mediating effect of the emotions generated during the use of the app by middle-aged and older people between their perceptions of the benefits of the cash subsidy and their continuous-use behavior:

**H4a:**  *Pleasure generated during the use of the Extreme Edition App by middle-aged and older people mediates the relationship between their perceptions of the benefits of cash subsidies and their continuous use behavior*.

**H4b:**  *The sense of worry generated during the use of the Extreme Edition App by middle-aged and older people mediates the relationship between their perceptions of the benefits of cash subsidies and their ongoing use behavior*.

## 3. Materials and Methods

### 3.1. Sample and Procedure

Our survey was conducted in mid-January 2024 with the assistance of Beyond Research Investigations to ensure the scientific validity of the study. As of 12 February 2024, about 1400 participants had completed the questionnaire. The questionnaires were anonymized to ensure the privacy of the participants and did not involve human experimentation or identifiable human data collection. We deleted the questionnaires found to have the following problems: (1) no previous contact with the Extreme Edition App; (2) age did not meet the requirements of this study; (3) did not pass the polygraph question test; and (4) the length of time completing the questionnaire was less than one minute. In the end, 1200 valid questionnaires were obtained.

Among them, 45.67% were male and 54.33% were female. The average age of the participants was 57 years, with a relatively small number of respondents aged 65 years and older, accounting for 2.92% of the total sample. The largest number of respondents was aged between 50 and 54 years old, specifically accounting for 33.83% of the total sample, while those aged between 55 and 59 years and those aged between 60 and 64 years accounted for a comparable proportion of the total sample at 31.17% and 32.08%, respectively. As far as this study is concerned, the age distribution of the sample is relatively balanced, and at the same time reflects the fact that the older age group may experience a decrease in their willingness to use the internet as they grow older [42]. From the results of the education level survey, 31.09% of the participants had a bachelor’s degree or above, which is in line with the contemporary era of middle-aged and older individuals who prioritized earning a living over education in their younger years. Thus, the proportion of those with higher education is relatively low.

In addition, we surveyed the average annual disposable income of the participants and found that 73.59% of the respondents had an average annual disposable income of no more than CNY 30,000, which is a lower-middle-income level. This is in line with the viewpoint of “The World Social Situation 2023” published by the United Nations, which stated that “in both developed and developing countries, older people are more likely than people of working age to live in relatively poor households”. Most respondents were from East China, a relatively economically developed region of the country. This also means that internet penetration is better in the region, and participants are more receptive to digital technology. At the same time, most participants came from third- and fourth-tier cities, while first- and second-tier cities accounted for only 14.08%. According to QuestMobile’s 2019 “Extreme Edition App” Insight Report [4], internet companies set “cash subsidies” in the Extreme Edition App mainly to compete for users in the shrinking market, i.e., third- and fourth-tier cities. Overall, the questionnaire survey sample was highly persuasive.

### 3.2. Measurement

In addition to demographic data collection, we also investigated participants’ perceptions of the benefits of the “cash subsidy”, their feelings of pleasure and worry about using the app, and their continued use of it. Participants were asked to use a five-point Likert scale (1 = Strongly Disagree, 5 = Strongly Agree) to select responses to different questions that corresponded to their situation. In addition, 10 middle-aged and older people who had been using the app for years and had successfully cashed in were invited to evaluate the questionnaire’s presentation. Experts from related professions were also invited to suggest modifications to the questionnaire, and adjustments were made according to the above feedback before entering the stage of officially releasing the questionnaire. The descriptive statistics and correlation coefficients of the key variables are listed in Table 1.

Herein, we treat the continuous use of the Extreme Edition App by middle-aged and older people as the dependent variable, and the cognition of the benefits of cash subsidy of the app, defined quantitatively by four stages of the addiction model: triggering use, taking action, variable rewards, and additional investment as the independent variable.

This study mainly examines the situation of middle-aged and older people’s use of digital products in the internet era, specifically the relationship between their perceptions of the benefits of cash subsidies and their continuous use of the Extreme Edition App. To explore the relationship more comprehensively, pleasure and worry were introduced as mediating variables to increase the explanatory power of the study.

**Extreme Edition App continuous-use behavior.** The field of information technology (IT) mainly includes technology acceptance research, the willingness to use a system, and the behavior of continuous use because both initial adoption and continuous use have a certain influence on IT. Initial adoption is the stage at which product markets are opened, and continuous use is the key to sustainable product development. Bhattacherjee (2001) was the first to break through the research framework of information technology/information systems (IT/IS) adoption theory. Based on expectation–confirmation theory, he constructed an expectation–confirmation model of IS continuance (ECM-ISC) and conducted empirical tests [43]. Subsequently, Limayem (2007) extended the dependent variable to continuous-use behavior based on the model [44], and Bhattacherjee (2008) also extended the ECM-ISC, realizing that focusing on predicting behavioral intention was not sufficient [45]. Referring to Bhattacherjee’s measurement items set for the construct of continuous-use behavior and combining the characteristics of the subjects in this study, a total of three items were set: (1) I use the Extreme Edition App frequently to get cash subsidies; (2) I have been using the Extreme Edition App to get cash subsidies for many years; and (3) I will continue to use the Extreme Edition App to get cash subsidies frequently (Cronbach’s alpha = 0.813).

**Cash Subsidy Benefit Awareness.** Cash subsidy refers to the rewards, subsidies, and other benefits gained by users of the Extreme Edition App. With the development of internet technology, internet enterprises increasingly realize that to acquire a large user base to use for their products, they need to have more active users to bring higher economic value. Cash subsidies are the “magic bullet” used by Chinese internet companies to increase users’ dependence on their products. To measure cash subsidies, this study introduces the four phases of the ”Hooked” addiction model as proposed by Nir Eyal, namely trigger, action, variable reward, and additional input [28], which, according to Nir Eyal, provide internet companies with operational strategies to maintain users’ habits. Based on the four stages of the addiction model, this study sets four dimensions of questions around “cash subsidy,” as follows:

**A. Trigger.** A total of four items were set up: (1) my acquaintances introduced me to using the Extreme Edition App, (2) seeing the advertisement for the Extreme Edition App, which says “you can earn money by watching videos” made me want to download and use it, (3) being able to get a cash subsidy made me want to use the Extreme Edition App, and (4) to reduce boredom, I will want to keep using the Extreme Edition App. (Cronbach’s alpha = 0.864).

**B. Action.** A total of three items were established: (1) I find it easy to use the Extreme Edition App to earn coins and withdraw cash, (2) I choose to use the Extreme Edition App to obtain the cash subsidy, and (3) overall, I want to get the cash subsidy, and it is easy to do (Cronbach’s alpha = 0.870).

**C. Variable Rewards.** A total of three items were established: (1) I am willing to use the Extreme Edition App consistently to obtain more cash subsidies, (2) obtaining more cash subsidies provides me a sense of accomplishment, and (3) when I see my friends obtaining cash subsidies, I would like to obtain such subsidies as well (Cronbach’s alpha = 0.840).

**D. Additional input.** A total of three items were established: (1) I am willing to invest my time and effort in the Extreme Edition App to receive the cash subsidy, (2) receiving the cash subsidy can make me more willing to invest my time and effort in the Extreme Edition App, and (3) I am willing to make purchases on the Extreme Edition App to receive more cash subsidies (Cronbach’s alpha = 0.842).

**Pleasure.** The pursuit of fun and entertainment often motivates individuals to take action. Heijden (2004) argued that in hedonic information systems, people are more concerned with the pleasure of using the system, and that the effect of perceived fun may be more pronounced than other variables [46]. Lin et al. (2005) also found that pleasure plays an important role in positively influencing whether or not a user will continue to visit a website in a study on the degree of confirmation of a user’s page expectations [36]. In their study of website usage behavior, Moon and Kim (2001) introduced pleasure into TAM and found that pleasure affects users’ attitudes toward using the internet [34]. Therefore, in this study, we refer to the scale items established by Lin et al. and Moon and Kim on pleasure and establish a total of three items: (1) The content of the Extreme Edition App makes me happy, (2) I feel that time passes very quickly when I use the Extreme Edition App, and (3) in general, completing tasks on the Extreme Edition App provides me pleasure (Cronbach’s alpha = 0.829).

**Worry.** According to the results of Block and Keller’s (1995) experiments on different health problems, it was found that when individuals are not sure if following advice will achieve the desired outcome, they will process things more deeply as a means to achieve inner peace. Negative frames of information are more persuasive than positive frames when individuals think deeply about an object [47]. During the use of the Extreme Edition App by middle-aged and older people, if they are not sure whether the risks associated with its use can be controlled, their sense of worry will affect their decision on whether to continue using the app. Therefore, this study referred to Block and Keller’s study and summarized two items: (1) I am concerned that there are certain unknown risks associated with the use of the Extreme Edition App and (2) I would be concerned if I did not have any friends or relatives around me who are also using the Extreme Edition App (Cronbach’s alpha = 0.756).

In addition, we tested the accuracy of the measurement construct (i.e., the validity of the scale) through a validity analysis. The Kaiser–Meyer–Olkin (KMO) test value in this study was 0.908, and the result of Bartlett’s test (X^2^ = 12323.771, df = 231, *p* < 0.001) was large and significant. The KMO value of the Extreme Edition App’s persistent-use behavior was 0.715, the KMO value of cash subsidy benefit perception was 0.754 (of which the KMO values of triggered use, taking action, variable rewards, and extra input were 0.829, 0.835, 0.727, and 0.719, respectively), the KMO value of pleasantness was 0.723, and the KMO value of worrying was 0.50. These findings indicate the good validity of the scales in our study.

### 3.3. Statistical Analysis

**Figure 1:**
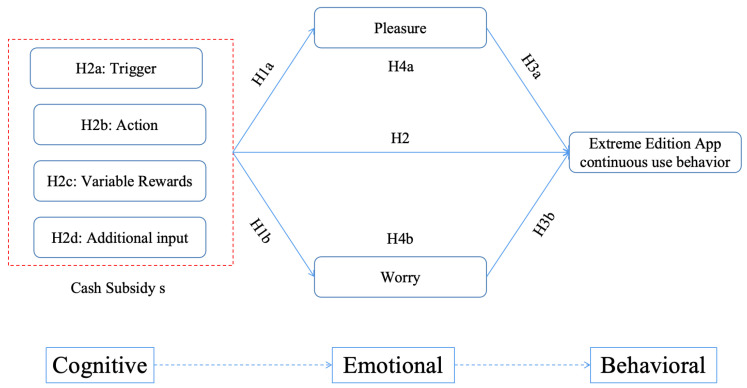
Cognitive–emotional–behavioral model.

Herein, we used middle-aged and older people’s continuous use of the Extreme Edition App as the dependent variable, and we used multiple regression to verify the relationship between the dependent variable and the independent variable, as well as the role of the mediating variable. Statistical analyses were conducted by first testing the model of the relationship between demographic variables (gender, age, and education) and dependent variables, and by testing the model of the relationship between perceptions of the benefits of cash subsidies and dependent variables. Second, the mediating effects of pleasure and worry on persistent-use behavior and perceptions of the benefits of cash subsidies were tested using the extended program Process v3.4 of SPSS 26.0 software. The specific research model is shown in Figure 1.

## 4. Results

### 4.1. Descriptive Results

After serial analysis of the study variables, we found that the study participants had a high level of awareness of the benefits of the four dimensions of the cash subsidy (Trigger to use M = 3.369; SD = 0.993) (Take action M = 3.354; SD = 0.996) (Multivariable reward M = 3.326; SD = 1.024) (Extra input M = 3.311; SD = 1.009). In addition, the level of behavioral performance of continuous use of the app was high (M = 3.348; SD = 0.981), indicating that the participants had continuous use of the app in real life, which made the study sample more representative. Among the gender (M = 0.457; SD = 0.498) control variables, women generated slightly (M = 3.319; SD = 1.023) more pleasure than men when using the app, and men produced slightly (M = 2.610; SD = 1.026) higher feelings of worry than women when using the app. This is consistent with the findings of previous studies on gender in rational and emotional thinking [48]. Overall, the independent, dependent, and mediating variables were statistically correlated.

### 4.2. Multiple Linear Regression

This study had a total of two parts using regression analysis. The first part focused on the cognitive, affective, and behavioral dimensions to explore the relationship between the dependent variable (Extreme Edition App continuous use behavior), the independent variables (four dimensions of cash subsidy benefit perceptions), and the mediating variables (feelings of pleasure and worry). In addition, the relationship between the four dimensions of perceptions of cash subsidy benefits of the independent variable and mediating variables pleasure and worry was addressed. To further explain the cognitive–behavioral relationship between the independent variables and the dependent variable, the second part introduced the mediating variables, i.e., positive feelings of pleasure and negative feelings of worry from the affective dimension, and it examined whether these feelings mediated the relationship between the independent variables and the dependent variable, respectively.

According to Model I in Table 2, we can see that all four dimensions of cash subsidy benefit cognition constructed by the four stages of the addiction model show significance, further verifying that there was some causal relationship between cognition and behavior. In Step 1, we tested the demographic variables, for which only the prediction of the income level variable was significant (β = 0.055, *p* < 0.05), implying that in this model, the higher the income level of the Extreme Edition App, the more consistently older users will use it. Based on these findings, the model for the demographic variables was significant: F(4,1195) = 2.458, *p* < 0.05, and the change in R2 was largely significant, but it accounted for only 0.8% of the behavioral variance.

In Step 2, four dimensions of perceptions of the benefits of cash subsidies were added based on the previous layer of demographic variable modeling. The results revealed that perceptions of the benefits of the cash subsidy significantly increased the variance explaining the participants’ continued use of the app (F(8,1191) = 53.407, *p* < 0.001), accounting for 25.6% of the behavioral variance. This showed that middle-aged and older people’s perceptions of the benefits of the Extreme Edition App’s “cash subsidy” were related to their continuous use of it, and H2a, H2b, H2c, and H2d were validated. We believed that the higher the willingness to use the Extreme Edition App triggered by the cash subsidy, the stronger the motivation and ability to take action by the cash subsidy and the more the variable subsidy brought the users’ needs to be fulfilled, while the more the cash subsidy triggered the additional investment in the Extreme Edition App, the more the middle-aged and old-aged users were likely to continue to use the Extreme Edition App. Overall, the more middle-aged and older people perceived the benefits of cash subsidies, the more sustainable their Extreme Edition App usage behavior was.

In addition to the verified cognitive–behavioral relationship, the relationship between emotion–behavior was reflected in Models II and III in Table 2. Step 1 of Models II and III were consistent with Model I, i.e., their demographic variables were both significant. Specifically, from Step 2 in Model I, 13.9% of the variance in continuous-use behavior was caused by the pleasure experienced by middle-aged and older people in the process of using the app (F(5,1194) = 41.215, *p* < 0.001), which was not a high percentage of the variance caused, but it indicated that the emotions generated by middle-aged and older people in the process of using the app and their continuous use of Extreme Edition App behavior were associated. H3a was valid; that was, the more pleasant the feelings middle-aged and older people felt when using the app, the more likely they would continue to use it. In Model III, the worrying feelings middle-aged and older people feel when using the Extreme Edition App (F(5,1194) = 30.454, *p* < 0.001) caused 10.5% of the variance in continuous-use behavior, which also implied that H3b was valid. That was, the more worried middle-aged and older people were during the use of the app, the less likely they would continue to use it.

According to Table 3a,b, at the cognitive–emotional level, cognition could influence the production of emotion. Specifically, in Models IV 1–2, we conducted a hierarchical multiple regression analysis of the perception of the benefits of cash subsidies using pleasure and worry as dependent variables. First, demographic variables (F(4,1195) = 0.727, *p* > 0.001) were tested in Step 1. However, none of them were significant in Models IV 1–2. This showed that emotions were generated as intrinsic motivation, independent of gender, age, education level, and income level.

Second, from Step 2, we found that 28.9% of the variance in the pleasure generated by middle-aged and older adults in the process of using the Extreme Edition App was caused by the four dimensions of the perception of the benefits of cash subsidies (F(8,1191) = 31.351, *p* < 0.001). In contrast, 20.6% of the variance in the middle-aged and older adults’ feelings of worry during the use of the app was caused by the four dimensions of the perception of the benefits of cash subsidies (F(8,1191) = 32.793, *p* < 0.001). These two variants indicated that there was a relationship between the perception of the benefits of the app’s “cash subsidy” and the emotions generated during the use of the app by middle-aged and older adults. H1a was valid, i.e., the higher the perception of the benefits of the cash subsidy, the more pleasure the middle-aged and older adults felt when using the app. Notably, H1b did not hold. Although the effect of the perception of the cash subsidy benefit on the generation of worry was significant, it was not a positive but a negative effect. This meant that the more the middle-aged and older people perceived the cash subsidy, the less the sense of worry generated during their use of the app. It could be seen that the various risks associated with the app were gradually ignored by middle-aged and older adult users in the process of their continuous use. On the one hand, the perceived benefits of cash subsidies may outweigh the perceived risks of using the app. On the other hand, middle-aged and older people may not have sufficient awareness of the risks associated with the app, which is noteworthy in future research.

The four models in the first part fully validated the cognitive–emotional–behavioral relationships and their interactions and influences. In the second part, we mediated intrinsic emotion in the extrinsic cognitive–behavioral relationship to examine whether the pleasure and worry generated by middle-aged and older people in the process of using the Extreme Edition App have a mediating effect between their perception of cash subsidy benefits and their behavior toward continuous use. Specifically, the samples were tested using the extended program Process v3.4 of SPSS 26.0 software, and the specific results are shown in Table 4.

From the data in Table 4, the mediating variables, pleasure and worry, did not include 0 between the lower limit confidence interval and upper limit confidence interval values of the independent variables and the dependent variables, i.e., there were significant mediating effects of pleasure and worry on the perception of the benefits of cash subsidies and the continuous-use behavior of the app by middle-aged and older users. According to the proportion of mediating effects, pleasure and worry were incomplete mediators. Specifically, regarding the mediating effect of cognition of cash subsidy benefits and middle-aged and older users’ continuous-use behavior of the Extreme Edition App, pleasure and worry senses mediated 15.7% and 10.7% of the effect, respectively. Therefore, H4a and H4b hold. Overall, to a certain extent, emotion mediated between cognition and behavior, and it promoted cognitive guidance of behavior.

## 5. Discussion

Combining the “attitude–behavior” model of the TPB and the cognitive–emotional–intentional model of psychology, this study proposes the “cognitive–emotional–behavioral” model, and, based on this model, it explores the digital life of middle-aged and older people. Specifically, the Extreme Edition App is taken as the research object, and the continuous-use behavior of the app with the function of “cash subsidy”, which is favored by middle-aged and older adults, is examined. The four dimensions of the cash subsidy were constructed using the four stages of the addiction model, and the relationship between middle-aged and older people’s awareness of the benefits of the cash subsidy of the Extreme Edition App, their feelings of pleasure and worry, and their continued use of the app was explored.

The results of the study show that, first, at the cognitive–behavioral level, there is a significant association between middle-aged and older people’s perceptions of the benefits of cash subsidies and their continuous use of the Extreme Edition App; the higher the perception, the more persistent the continuous use behavior. Second, at the affective–behavioral level, the pleasure experienced during the use of the app by middle-aged and older people significantly positively affects their sustained-use behavior, while the worry generated significantly negatively affects their sustained-use behavior.

Finally, at the cognitive–emotional level, middle-aged and older people’s perceptions of the benefits of cash subsidies are significantly linked to the emotions experienced by their use. The richer and deeper the cognition, the richer and clearer the pleasure experienced, and interestingly, the fewer and vaguer feelings of worry were generated. In addition, to confirm the relationship between cognitive–emotional–behavioral patterns, we introduced emotion as a mediating variable between cognitive–behavioral relationships. The results revealed significant mediating effects for pleasure and worry, serving as incomplete mediators.

Cognitive–behavioral findings are consistent with the results of previous research. Pieniak et al. (2010), in their investigation of factors influencing consumers’ organic food-consumption behavior, pointed out that subjective knowledge is an important factor explaining the consumption behavior of organic vegetables and is more predictive of an individual’s behavior than objective knowledge [49]. Middle-aged and older people in contemporary China have experienced the transition from being fed to being rich, and their subjective knowledge of money will be more profound. The cash subsidy function in the app advocates the slogan “You can make money by watching video ads”, which further stimulates middle-aged and older people’s perceptions of economic value. The fact that they can obtain cash rewards while being entertained is the key point for them to be glad to use the APP. However, what we need to pay attention to is that the Extreme Edition App attracts middle-aged and older-age people with its money-making features on the surface. However, in fact, it contains the hidden worry that middle-aged and older-age people’s data will be “harvested” by the traffic.

Emotions originate from within the individual and arise at both the cognitive and behavioral stages. According to the socio-emotional selectivity theory, people are more willing to maintain fewer relationships as they grow older, and middle-aged and older people spend more time and energy maintaining a few close relationships [50]. On the one hand, middle-aged and older people have smaller social circles after retirement. On the other hand, they lack social resources to initiate new relationships. However, based on digital technology, internet products bring equal socialization opportunities and resources to these people to a certain extent. They can watch video entertainment on the app and obtain cash subsidies at the same time, which largely satisfies their economic needs and fills their spiritual emptiness, and they can garner richer emotions during the process of using the app.

Of all the hypotheses in this study, the hypothesis that did not hold appeared in examining the impact of middle-aged and older adults’ perceptions of the benefits of cash subsidies on the emotions generated during their use of the app. We found that their perceptions of the benefits of cash subsidies positively influenced positive pleasure generation and negatively influenced negative worry generation. For these people, the act of engaging with new technology means that they can defend themselves against the risks that the act may bring. At the same time, the positive feedback received in the process of continuous use, such as receiving pleasure and additional subsidies, further weakens their perceptions of risk, which, in turn, reduces their emergence of a sense of worry. It is undeniable that these people have been labeled as marginalized internet users. However, they also continued to improve their digital literacy and skills by using the Extreme Edition App and subsequently became more confident in resisting unknown risks. In addition, as a mediating variable, emotion amplifies respondents’ perceptions of the benefits of cash subsidies. To consistently gain more pleasure and reduce worry, users adopt the behavior of using the app more consistently.

Overall, we can see that the three dimensions of cognitive, affective, and behavioral are not linear or independent of each other but rather complementary and mutually influential. Blair et al. (2023) explored the cognitive, affective, and behavioral mechanisms of the social network effect, and the study pointed out that when a couple confirms that their relationship is supported, it is perceived to be supported in the cognitive aspects of their affection-sharing as well. Based on this, they engage in more affection-sharing behaviors and have a richer sense of well-being [51]. Understanding the current digital lives of middle-age and older people from the cognitive, emotional, and behavioral dimensions involved in their continuous use of the Extreme Edition App enriches the research horizon.

Finally, we also found that income level has a significant effect on the continued use of the Extreme Edition App by middle-age and older adults. Although the explanatory power is low, it provides a glimpse into the inner need for a sense of security that income brings to people who have left the workforce. Widening labor market gaps may increase inequality as people age. People aged 50 and above have lower life satisfaction, fewer social interactions, and less social support than those aged 15–49 [52]. In the context of agism, people aged 50 and over also report a lack of agency or control over their lives and feel that they are not treated with dignity and respect [53]. Future researchers may be able to further understand the dimensions that influence middle-aged and older adults’ perceptions of adapting to digital life from the perspective of perceived dignity.

## 6. Strengths and Limitations

The main theoretical contribution of this study is to organically combine the “attitude–behavior” logic of the TPB with the “cognitive–emotional–intentional” model of classical psychology and to propose a “cognitive–emotional–behavioral” model to explore the specific situation of Chinese middle-age and older Chinese people’s digital lives. This study also proposes a “cognitive–emotional–behavioral” model to explore the specifics of the digital lives of these people in China. The findings of this study demonstrate that cognition, emotion, and behavior have a mutually reinforcing relationship. Specifically, the deeper the group’s perceptions of the benefits of cash subsidies, the richer the pleasure generated by their use of the app, the weaker the worry, and the more persistent their behavior of continuous use of the app. Overall, this is an innovation in knowledge mapping of TPB and psychological modeling. In addition, this study applies the addiction model to the study of usage behavior, expanding the empirical research mapping of the addiction model on user experience.

Examining the behavior of this group’s Extreme Edition App usage can, on one hand, prompt internet platforms to shoulder their due social responsibility. On the other hand, it can be of guiding significance to improve the internet survival literacy of middle-age and older people. It helps them, as well as relevant government departments and organizations caring for older people, to further recognize how to enjoy old age in a more meaningful and dignified way in the internet era.

The limitations of this study are: first, the results of exploring the digital life situations of middle-aged and elderly people are mainly applicable to the Chinese region. Perhaps our findings are informative to a certain extent for other countries, but their explanatory power is relatively limited in the global dimension. Second, despite the increasing internet penetration in China, complete equality in the use of digital technology cannot be guaranteed at present. From the 53rd Statistical Report on Internet Development in China released by CNNIC, China’s internet penetration rate reached 77.5% as of December 2023, and the phenomenon of the digital divide between urban and rural areas still affects the study [4]. Finally, only a relatively representative cross-section of the digital life of the middle-age and elderly group was selected for this study. If we want to reflect the digital life of this group more comprehensively, we need to cut more scenes to build a complete framework.

## 7. Conclusions

This study combines the “attitude–behavior” logic of the TPB and the “cognitive–emotional–intentional” model of psychology and proposes a “cognitive–emotional–behavioral” model. In this study, we explored the relationship between middle-age and older people’s perceptions of the benefits of the Extreme Edition App’s cash subsidy function, the emotions of pleasure and worry that arise from the use of the app, and their behavioral intention to continue to use it. The four stages of the addiction model (trigger, action, reward, and engagement) were used to quantitatively express the cognitive variables of the benefits of the cash subsidy function, further enriching the scientific and innovative nature of the research framework.

The study found that first, middle-age and older people’s perceptions of the benefits of cash subsidies and their pleasure (or worry) in using the Extreme Edition App positively (or negatively) influenced their continued use of the app. Second, this group’s perceptions of the benefits of cash subsidies significantly and positively affected their feelings of pleasure and negatively affected their feelings of worry during the process of using the app. Finally, pleasure and worry in the process of using the app played an imperfect mediating role between this group’s perceptions of the benefits of the cash subsidy and their continued use of the app. Overall, cognition, emotion, and behavior explain well the state of this group in the continuous use of the app, and all three factors influence and complement each other. This provides a reliable reference point for enterprises and the government to help middle-aged and older people truly integrate into digital life and enjoy digital dividends.

## Figures and Tables

**Table 1:** Descriptive statistics of key variables.

Variable (V)			M	D
V1 (the cognition of the benefits of cash subsidy of the app)	V1-1	Triggering use	3.369	0.993
	V1-2	Taking action	3.354	0.996
	V1-3	Variable rewards	3.326	1.024
	V1-4	Additional investment	3.311	1.009
V2		Pleasure	3.319	1.023
V3		Worry	2.610	1.026
V4		Continuous use of the Extreme Edition App	3.348	0.981

**Table 2:** Multiple linear regression of the continuous use behavior of middle-aged and elderly users of Extreme Edition App.

Model I				Model II				Model III			
Step	Variable Entered	β	β	Step	Variable Entered	β	β	Step	Variable Entered	β	β
1	Gender (male = 1)	−0.103	0.076	1	Gender (male = 1)	−0.103	−0.088	1	Gender (male = 1)	−0.103	−0.089
	Age	0.006	−0.037		Age	0.006	0.000		Age	0.006	−0.021
	Education	0.002	−0.017		Education	0.002	−0.005		Education	0.002	−0.011
	Income	0.055 *	0.051 *			0.055 *	0.043 *		Income	0.055 *	0.053 *
2	Triggering use		0.203 ***	2	Pleasure		0.358 ***	2	Worry		−0.310 ***
	Taking action		0.170 ***								
	Variable rewards		0.123 ***								
	Additional investment		0.174 ***								
	N	1200	1200		N	1200	1200		N	1200	1200
	R^2^/adjusted R^2^	0.005/ 0.005	0.259/ 0.259		R^2^/adjusted R^2^	0.005/ 0.005	0.144/ 0.144		R^2^/adjusted R^2^	0.005/ 0.005	0.109/ 0.109
	ΔR^2^	0.008	0.256		ΔR^2^	0.008	0.139		ΔR^2^	0.008	0.105
	ΔF	2.458 *	103.512 ***		ΔF	2.458 *	3469.308 ***		ΔF	2.458 *	141.280 ***
	Model F	2.458 *	53.407 ***		Model F	2.458 *	1505.532 ***		Model F	2.458*	30.454 ***

* *p* < 0.05, *** *p* < 0.001; The dependent variable is Extreme Edition App continuous-use behavior.

**Table 3:** (**a**) Multiple linear regression of the feeling of pleasure. (**b**) Multiple linear regression of the feeling of worry.

**(a)**			
**Model IV−1**			
**Step**	**Variable Entered**	**β**	**β**
1	Gender (male = 1)	−0.044	−0.013
	Age	0.017	−0.033
	Education	0.022	0.045
	Income	0.033	0.020
2	Triggering use		0.214 ***
	Taking action		0.143 ***
	Variable rewards		0.181 ***
	Additional investment		0.204 ***
	N	1200	1200
	R2/adjusted R2	−0.001/−0.001	0.287/0.287
	ΔR2	0.002	0.289
	ΔF	0.727	121.682 ***
	Model F	0.727	61.351 ***
*** *p* < 0.001; The dependent variable is the feeling of pleasure.			
**(b)**			
**Model IV−2**			
**Step**	**Variable Entered**	**β**	**β**
1	Gender (male = 1)	0.047	0.021
	Age	−0.086	−0.044
	Education	−0.044	−0.024
	Income	−0.007	−0.003
2	Triggering use		−0.188 ***
	Taking action		−0.155 ***
	Variable rewards		−0.135 ***
	Additional investment		−0.152 ***
	N	1200	1200
	R2/adjusted R2	−0.002/−0.002	0.203/0.203
	ΔR2	0.002	0.206
	ΔF	0.546	77.444 ***
	Model F	0.546	39.065 ***
*** *p* < 0.001; The dependent variable is the feeling of worry.			

**Table 4:** The mediating effects of pleasure and worry mediating variables.

Intermediate Variable	Total Effect of X on Y	Indirect Effect (s) of X on Y			Proportion of Intermediary Effects to Total Effects (%)
		Effect	LLCI	ULCI	
Pleasure	0.669	0.105	0.0643	0.1468	15.7%
Worry	0.669	0.0727	0.0398	0.1073	10.9%

The dependent variable is Extreme Edition App continuous-use behavior. The independent variable is cash subsidy benefit awareness.

## Data Availability

The data presented in this study are openly available by contacting the corresponding author.
